# Human-centricity in AI governance: A systemic approach

**DOI:** 10.3389/frai.2023.976887

**Published:** 2023-02-14

**Authors:** Anton Sigfrids, Jaana Leikas, Henrikki Salo-Pöntinen, Emmi Koskimies

**Affiliations:** ^1^VTT Technical Research Centre of Finland Ltd, Espoo, Finland; ^2^Faculty of Information Technology, Cognitive Science, University of Jyväskylä, Jyväskylä, Finland; ^3^Faculty of Management and Business, Administrative Sciences, Tampere University, Tampere, Finland

**Keywords:** artificial intelligence, human-centered, ethics, governance, collaboration, social sustainability, AI for the common good

## Abstract

Human-centricity is considered a central aspect in the development and governance of artificial intelligence (AI). Various strategies and guidelines highlight the concept as a key goal. However, we argue that current uses of Human-Centered AI (HCAI) in policy documents and AI strategies risk downplaying promises of creating desirable, emancipatory technology that promotes human wellbeing and the common good. Firstly, HCAI, as it appears in policy discourses, is the result of aiming to adapt the concept of human-centered design (HCD) to the public governance context of AI but without proper reflection on how it should be reformed to suit the new task environment. Second, the concept is mainly used in reference to realizing human and fundamental rights, which are necessary, but not sufficient for technological emancipation. Third, the concept is used ambiguously in policy and strategy discourses, making it unclear how it should be operationalized in governance practices. This article explores means and approaches for using the HCAI approach for technological emancipation in the context of public AI governance. We propose that the potential for emancipatory technology development rests on expanding the traditional user-centered view of technology design to involve community- and society-centered perspectives in public governance. Developing public AI governance in this way relies on enabling inclusive governance modalities that enhance the social sustainability of AI deployment. We discuss mutual trust, transparency, communication, and civic tech as key prerequisites for socially sustainable and human-centered public AI governance. Finally, the article introduces a systemic approach to ethically and socially sustainable, human-centered AI development and deployment.

## 1. Introduction

The significance of artificial intelligence (AI) technologies lies in their ability to surpass data processing limitations for the benefit of humans (Deguchi et al., 2020; Babic et al., 2021; Crawford, 2021). Mass data, predictive analytics, and AI can be used to find new ways to make people's lives easier, contribute to more efficient public services, and improve human performance (Wirtz and Muller, 2019; Wirtz et al., 2019; Mikalef et al., 2021; Samuel et al., 2022). AI technologies present potentially substantial benefits to individuals, businesses, and society (Brynjolfsson and Mcafee, 2017), but there is considerable debate about the problems and risks involved (e.g., Floridi et al., 2018), about who reaps the benefits, and who is negatively affected (Zuboff, 2019; Crawford, 2021; WRP, 2021), how to weight different risks, benefits, interests, and values in decision-making (Sigfrids et al., 2022; Wirtz et al., 2022), and about the means to secure the common good and human flourishing with AI (Floridi et al., 2020; Stahl et al., 2021). These questions are central in developing public governance of AI, which aims to steer AI development and deployment to mitigate the risks and maximize the benefits of AI solutions to society.

Governance of AI is itself a complex, developing field that contains different positions and frames regarding the relevant governance concerns (e.g., Taeihagh, 2021; Wirtz et al., 2022). It “*includes various frameworks, processes, and tools designed to maintain and promote cooperative possibilities to formulate shared values for AI, as well as to make and implement decisions regarding desirable directions in the development and use of AI*” (see also, Dafoe, 2018; Sigfrids et al., 2022, p. 3–4). A central question for public governance is how to develop frameworks and institutional governance arrangements that can sustain a legitimate jurisdiction while fostering human-centered values amidst contesting values, normative differences, and complex trade-offs among nations, corporations, social groups, and individuals.

Literature on developing public AI governance (Taeihagh, 2021; Sigfrids et al., 2022; Wirtz et al., 2022) and emerging technology governance more generally (Kuhlmann et al., 2019; Lehoux et al., 2020) emphasize increasing horizontal coordination and stakeholder and public engagement, what we term *inclusiveness*, in decision-making. Public administrations can by such means improve the quality and legitimacy of public decision-making, empower citizens, and increase their trust in the public administration (OECD, 2017, 2020). Inclusiveness also supports a more systemic, comprehensive informational basis for public decision-making, decreases informational asymmetries (Sigfrids et al., 2022), and increases the flexibility of public governance (Kuhlmann et al., 2019; Lehoux et al., 2020). Such governance practices, combined with mechanisms that guide AI education and research to a multidisciplinary direction (Auernhammer, 2020; Salo-Pöntinen and Saariluoma, 2022), increase the possibility to include expectations and values of communities affected by the development and uptake of AI solutions already in the design of said solutions (Owen et al., 2013; Jasanoff, 2016; IEEE, the IEEE Global Initiative on Ethics of Autonomous and Intelligent Systems, 2019). In addition, they improve the detection of societal challenges and possibilities of AI development and deployment (Jasanoff, 2016; Crawford, 2021; Salo-Pöntinen and Saariluoma, 2022). If realized, both perspectives have a positive impact on the general legitimacy of AI development and use.

In parallel with the identification of ethical requirements for AI development, human-centered AI (HCAI^1^) has emerged as a key concept and goal in policy papers aiming to develop public governance of AI (AI HLEG, 2019; Salo-Pöntinen and Saariluoma, 2022). Its promise for framing governance goals and practices lies both in improving human performance and supporting better human-technology interaction (Riedl, 2019; Lepri et al., 2021; Shneiderman, 2022), and in a commitment of the public governing institutions to steer technology development and deployment to serve humanity and the common good (AI HLEG, 2019). The latter position, which emphasizes national public administrations' role in steering technology in a desirable direction for humanity while minimizing the risks to society, we call the emancipatory viewpoint to technology governance (Frey et al., 2021).

We argue in this perspective paper that current uses of HCAI in policy documents risk downplaying its promise of creating desirable, emancipatory technologies that promote human wellbeing and the common good. This is due to three factors. *First*, HCAI, as it appears in policy discourses, is the result of aiming to adapt the concept of human-centered design (HCD) to the public governance context of AI but without proper reflection on how it should be reformed to suit the new task environment (Auernhammer, 2020; Salo-Pöntinen and Saariluoma, 2022). The application of HCD has faced similar criticism of unreflective adoption when it has been introduced as part of new design paradigms (Bannon, 2011). As a result, it fails to consider broader political, ethical, and legal issues that public administrations need to account for. *Second*, the concept is mainly used in reference to realizing human and fundamental rights, which are necessary, but not sufficient for emancipatory goals of human flourishing. Third, the concept is used in an ostensibly established manner in policy and strategy discourses, but it proves to be ambiguous when scrutinized more closely. As a result, it becomes unclear how it should be operationalized in governance practices to achieve its goals.

We argue that to enable technological emancipation, it is essential that the public governance and design of AI are socially sustainable, i.e,. based on public governance that is inclusive and comprehensive in a way that puts the societal, economic, and environmental impacts, and the needs and values of people and communities at the center of AI governance and deployment (Sigfrids et al., 2022; Wilson and Van Der Velden, 2022). We propose that human-centered AI thinking can be incorporated into public governance approaches by holistically considering viewpoints and inputs from individuals with different backgrounds, interests, and values (e.g., Sigfrids et al., 2022), the expectations of affected communities (Owen et al., 2013; Jasanoff, 2016; IEEE, the IEEE Global Initiative on Ethics of Autonomous and Intelligent Systems, 2019), and accounting for contextual (e.g., Saariluoma et al., 2016; Salo-Pöntinen, 2021) and societal (e.g., Jasanoff, 2016; Crawford, 2021) dimensions in technology design. This ideal of human-centricity can be further elaborated by defining it as containing three perspectives: user-centeredness,^2^ community-centeredness, and society-centeredness. We argue that enabling emancipatory technology development requires integrating all three perspectives. Using this framing to construct HCAI as a concept for technology emancipation, we argue that this integration of perspectives fails in the tradition of HCD and in the way the concept has been applied in AI policy and strategy papers. Considering the major impact of AI on everyday life, it is the task of democratic governments to enable citizens and impacted stakeholders and communities to partake in common debates and decision-making about the uses of AI. Such an approach to governance can be supported by building mutual trust, transparency, and technology solutions that lay the grounds for informed communication and dialogue between different stakeholders (Buhmann and Fieseler, 2022; Stahl, 2022; Wilson and Van Der Velden, 2022).

In this perspective article, we highlight key prerequisites for publicly governing AI systems in a human-centric manner. We first discuss the potential pitfalls of current uses of the concept in policy papers, and present trust, transparency, and communication as main elements for governing socially sustainable human-centered AI. Second, we show that the AI governance literature emphasizes the importance of inclusive, collaborative policies in contributing to ethical and sustainable AI development and discuss novel collaboration tools that could be used to facilitate broad stakeholder deliberation. To synthesize the discussion, we propose a systemic approach to governing ethically and socially sustainable, human-centered AI development and deployment.

## 2. Human-centricity in the public governance of AI

HCAI has become an important concept and goal in research and policy papers on how to steer and design AI to support the realization of beneficial aspects of AI to individuals and society (Salo-Pöntinen and Saariluoma, 2022; Shneiderman, 2022). In general terms, there are two approaches to human-centered AI: one originating from user-centered technology design and the other representing its use in policy papers.

From a user-centered design perspective [also known as human-centered design (HCD)], HCAI seeks to improve human-technology interaction and human performance by focusing on actual human capabilities, needs, and values and ensuring that ethical principles are met in the design of AI products. Researchers applying the concept emphasize the importance of AI system reliability and safety, and ethical principles such as fairness, accountability, interpretability, and transparency in the governance of AI (Riedl, 2019; Lepri et al., 2021; Shneiderman, 2022).

National AI strategies and policy papers use the concept more broadly. Here human-centricity has emerged as a central, but multivocal concept that is mainly used to bundle together a set of ethical and human rights principles as a basis for an AI strategy, goal, or vision (Salo-Pöntinen and Saariluoma, 2022).^3^ One of the leading documents for the EU's AI strategy, the EU's Ethics Guidelines for Trustworthy AI (AI HLEG, 2019, p. 4), states that AI systems “*need to be human-centric, resting on a commitment to their use in the service of humanity and the common good, with the goal of improving human welfare and freedom*”. Furthermore, it defines human-centric AI as an approach that “*strives to ensure that human values are central to the way in which AI systems are developed, deployed, used and monitored, by ensuring respect for fundamental rights*” (AI HLEG, 2019, p. 37).

In contrast to the user-centric technology design perspective (e.g., Shneiderman, 2022), the EU's Ethics Guidelines seem to broaden the goals of human-centered AI from improving human performance to a concept for serving the common good, increasing wellbeing, and enabling respect for human and fundamental rights. This viewpoint, which we call the emancipation viewpoint, changes the level of ambition and expected impact carried by the concept. We call out three problems for the emancipation viewpoint in the way HCAI has been used in policy papers.

Firstly, in light of the ambition and goals invested in the HCAI concept in policy documents, the EU's Ethics Guidelines concept of HCAI risks a failed adaptation of the main ideas behind HCD to a public governance context. The guidelines should not be interpreted as referring to the tradition of user-centered design, because the traditional contexts and prerequisites of human-centricity in technology development have been formulated to acknowledge immediate technology use situations, and not necessarily to consider wider perspectives that are necessary for the emancipatory viewpoint. AI design is not only a multi-technological effort; it also involves ethical, social, psychological, economic, political, and legal aspects, and is likely to have a profound impact on society (Lucivero, 2016; Frey et al., 2021). Considering this, the traditional use of the human-centric concept in policy papers risk leading the discussion of technology development and design thinking in a reductive direction. It truncates thinking and aligns it with the human-centered design standards, thus emphasizing the perspective of a human being as “a user”, and people as “user groups”. Placing citizens in the position of users narrows and isolates the perspective from the broader and more indirect political and environmental conditions and impacts of AI systems, such as the power structures of digital economies, the environmental impact of material production of digital technologies, and the impact on work-life (Crawford, 2021), or the significant risks of social media for mental health (Boer et al., 2020; Rathje et al., 2021) and democracy (Epstein and Robertson, 2015; Nemitz, 2018; Ledger of Harms, 2021). In contrast, a shift from a user-centered view to a human-centered perspective would in principle consider the agency of citizens and communities in terms of their participation in collective decision-making that steers, for example, the activities of large technology companies or AI use in public services.

Secondly, policy papers run a risk of additionally narrowing the viewpoint if they use HCAI mainly as a reference for respecting human and fundamental rights (Salo-Pöntinen and Saariluoma, 2022). In terms of the emancipation viewpoint, this is problematic since human rights are barely “*minimum necessities for respecting human dignity but do not function as a holistic approach for defining human flourishment*” (Salo-Pöntinen and Saariluoma, 2022, 7). While necessary and immensely important, human and fundamental rights as the basis for public governance are not in themselves sufficient to support realizing the benefits of AI (Jasanoff, 2016; Canca, 2019).

The third problem facing the emancipation viewpoint to human-centric AI governance is that in the absence of uniform definitions, the HCAI concept lacks essential meaning in policy papers and thus has little operational value to public governance mechanisms. This is however a more general concern for ethical and responsible AI governance since, despite guidelines and recommendations, the ethical and responsibility principles that could contribute to a socially sustainable, human-centric AI, have not been successfully implemented in practice (Dignum, 2019; Hagendorff, 2020; Raab, 2020; Schiff et al., 2021). The risk is the so-called ethics washing and legitimation of the status quo by reference to the existing ethics guidelines, but with no significant impact. A revision of what the HCAI concept entails in terms of its expected impacts and objectives is thus a reasonable requirement in framing and countering new challenges and promoting the promises of AI.

We claim that the unreflective and ambiguous use of the HCAI concept in policy papers, and the lack of its operationalization, risk downplaying the emancipatory role and the desirability of technology and innovations, thus hindering AI technologies' potentially significant benefit for humanity. If the broader political and socio-technical impacts on different groups of people are not taken into consideration and operationalized in human-centric technology design and governance, there is a danger that AI services will primarily be built and used to prioritize the needs and interests of technology owners and designers rather than the interests of humankind (Zuboff, 2019; Crawford, 2021; Frey et al., 2021; De Cremer et al., 2022). To realize broader sustainability goals than simply user-centered design as in enhancing the quality of human-technology interaction, HCAI could benefit from a focus on the societal preconditions of humane technology and the common good that are rooted in sustainable AI governance. In other words, the user-centered perspective should be compounded with the community- and society-centered perspectives to enable emancipatory technology development.

Placing the focus on social sustainability requires considering the systemic implications of technology on humans and societies (UN, 2012). This means considering how the complex and dynamic interplay between technology, operators, users, citizens, and society operates, how these agents are influenced by technology, and how a new AI culture changes human ecosystems. It also means accounting for the short- and long-term, direct and indirect economic, social, and environmental opportunities, problems, and risks of developing and deploying AI systems. Concerning governance procedures, this perspective means considering who and what viewpoints frame the development strategies and practices, and to what degree the process is inclusive, democratic, and transparent.

Socially sustainable AI governance builds on ensuring diverse and inclusive participation in decision-making, building trust, supporting communication, and common-meaning formulation (Wilson and Van Der Velden, 2022). Enabling meaningful stakeholder and citizen inclusion in decision-making is a major theme in the literature proposing solutions to the problem of ethically governing AI deployment. A systematic review of the theme (Sigfrids et al., 2022) indicates that public AI policy decisions should be made through a comprehensive and inclusive approach to ensure that they are based on an understanding of short- and long-term ethical and socio-technical implications. Here inclusive stakeholder participation ensures that decisions are based on multiple viewpoints and broad expertise and that decisions are legitimate and appropriate to local contexts.

To function, the calls for sustainable governance and inclusive decision-making require a certain amount of mutual trust, communication, and transparency (Buhmann and Fieseler, 2022; Stahl, 2022), which is made possible by aligning technical and organizational practices of data and service ecosystems to safety and ethical standards. Maintaining and building possibilities for safe and robust AI systems, and supporting transparency and explainability of AI models enables accountability, which is a necessary trust-building element needed for organizations and industries deploying and developing AI (AI HLEG, 2019; Sutrop, 2019; Shneiderman, 2022). Aligning AI development with the community- and society-centered perspectives means stakeholder involvement, responsiveness to their viewpoints (von Schomberg, 2011; Owen et al., 2013), and open cross-disciplinary communication and dialogue on uncertainties and general concerns about AI (Blankesteijn et al., 2014; Stahl et al., 2017; Floridi et al., 2018; Dignum, 2019).

### 2.1. Prerequisites for trust and informed public dialogue

The concept of earning trust in the governance of technology was first introduced at the World Economic Forum Global Future Council (GFC) in 2016 (WEF, 2016). Since then, several parties have raised trust as an essential element in the use of AI (see IBM, 2018; AI HLEG, 2019; G20, 2019; OECD, 2019; USNSTC, 2019; European Commission, 2020; UNESCO, 2021). References to trust in AI include the trustworthiness of research, trustworthy AI designers and developers, trustworthy organizations, trustworthy design principles and algorithms, and the responsible deployment of AI applications. The draft regulation of the European Commission (2021) called *Laying Down Harmonized Rules on Artificial Intelligence* (AI Act), released in April 2021 seems to suggest that current technological infrastructures are untrustworthy, and that regulation is necessary to increase trust in both AI systems and society in general (Bodó, 2021).

Understanding the prerequisites for trust is important for societies, communities, and cultures creating social rules for deploying AI, because trust is critical in the social capital that holds society together (Bodó, 2021), and a prerequisite for the sustainable data economy and use of AI. Sutrop (2019) distinguishes two forms of trust: trust in developers of AI services creates social trust, whereas reliable processes, structures, values, and culture build non-personal systemic trust.

Building and maintaining trust involves social and technical structures that ensure accountability of AI systems even in complex use contexts where the impacts are hard to predict. Trust is always tied to a context (Langer et al., 2022) and must be considered in terms of the local setting, institutions, stakeholders, and technologies, within which AI is used. This means that it is essential to consider organizational processes, structures (Zicari et al., 2021), and technical components in terms of how they enable trust in AI systems (e.g., Tsamados et al., 2022). Thus creating trustworthy AI systems on the technical and organizational level demands technical robustness, explainability, transparency, traceability, and accountability (AI HLEG, 2019; Floridi and Cowls, 2019; Gillespie, 2019; Kingsman et al., 2022).

In other words, the main criteria for building trust is that humans must to some extent be able to understand how the AI system functions and how the AI decisions are arrived at. *Developers* must be able to explain how and why a system acts the way it does. *Applications* must include explanations of how undesirable effects will be detected, stopped, and prevented from reoccurring. Trustworthy AI systems are a prerequisite for critical public scrutiny (Dignum, 2019) and informed public debate (Buhmann and Fieseler, 2022). However, also multidisciplinary dialogue and a holistic understanding of the different perspectives on the impacts of AI are needed to build more solid foundations for mutual trust and possibilities for communication and collective decision-making.

### 2.2. Mutuality supports interdisciplinary communication and collective decision-making

The design and deployment of AI brings together stakeholders from different disciplines and institutions with different institutional logics. This leads to a situation where AI governance is characterized by social interaction and interdependence (Thibaut and Kelley, 1959) which challenges the existing organizational arrangements and control hierarchies and makes it necessary to discuss mutuality as a phenomenon that shapes AI governance. Yeoman (2019) suggests that mutuality is an ethical organizing principle in which collective social and environmental wellbeing is created through mutual interdependence between stakeholders. Thus, building an ethically sustainable society is “dependent upon the extent to which mutuality is designed into organizational purpose, structures, and processes” (Yeoman, 2019, p. 92). Mutuality can hence be seen as an approach to AI governance (Koskimies and Kinder, 2022) in the sense that making decisions about the utilization and control of AI should be based on mutuality between stakeholders and the consideration of various stakeholder and citizen interests, values, and perspectives (Levi and Stoker, 2000; Owen et al., 2013).

Mutuality is based on inclusiveness. Leng (2016) argues that mutuality involves equity, autonomy, solidarity, and participation. Mutuality supports user engagement at all design and decision stages thus building foundations for trust (Koskimies and Kinder, 2022). When multiple databases, information- and decision systems are brought together, the flourishing of human autonomy in the multiagent collaboration is dependent on the commitment of stakeholders to detect and regard all stakeholder needs (Koskimies and Kinder, 2022).

To enable mutual decision-making, involved stakeholders must commit to learning about the values of different stakeholders (Koskimies et al., 2022). Here communication is necessary for sharing social experiences and turning attention toward what is desirable to improve quality of life. For example, Koskimies and Kinder (2022) argue that communication about AI development in the central government is limited by the inability to find a shared language between stakeholders, and because the content of communication relates more to the technical aspects of AI or managerial goals such as costs and efficiency than ethical impacts and values. In other words, stakeholders must possess certain cognitive, prosocial, and cultural skills to participate in cooperative communication and to formulate joint intentions and goals needed for collective problem-solving (Yeoman, 2019). Hence, it is important to foster the communicative actions of stakeholders that build mutual trust and eventually inclusive and socially sustainable decision-making in AI adaptation.

Mutuality, which describes the trust, interdependence, and reciprocity between stakeholders, then becomes one of the necessary organizing principles of collective interactions of stakeholders contributing to decision-making. An inclusive form of governance should enable the integration of the points of view of different fields of the humanities and social sciences (Werthner et al., 2022), and technical sciences in an enlightened dialogue. Multidisciplinary communication could enable a more holistic and systemic understanding of human, community, and societal perspectives and values to be accounted for in public decisions on technology development (Nussbaum, 2010; Saariluoma et al., 2016; Werthner et al., 2022). Enabling multidisciplinary dialogue, awareness-building, and learning among AI experts, humanities and social science academics, businesses, and the general public would increase awareness of AI ethics and foster informed public debate, and potentially ethical self-regulation capabilities among businesses (Floridi et al., 2018; Donahoe and Metzger, 2019; Truby, 2020).

## 3. Collaborative tools contribute to inclusive decision-making

In the interactive and mutually responsible process of AI development, communication is often challenged by strong knowledge boundaries and information asymmetries between the agents involved, which undermines trust and democratic governance (Buhmann and Fieseler, 2022; Stahl, 2022). Miscommunication can lead to oppression, exploitation, restrictions on freedom, and disinformation, and can prevent transparency and access to relevant information. Thus, communication between citizens and decision-makers should be explicit. The development of an AI society requires principles governing communication activities (Habermas, 1981), as well as a trustworthy governance system, and entails policies that support democratic processes and transparency when tackling the societal challenges of AI use (Nieminen and Ikonen, 2020). This is a multi-level governance challenge: there must be a shared and coordinated understanding across various social and administrational sectors on how AI policy should be coordinated and AI deployment regulated. To be able to counter the local and global ethical challenges of developing and deploying AI technology (Coeckelbergh, 2020), public administrations need to develop new practical governance frameworks and tools to support the formation of a shared understanding of the challenges, solutions, and values to be pursued in steering AI use and development.

To pursue this goal, many international initiatives for AI governance emphasize multi-stakeholder collaboration and highlight the importance of incorporating a wide variety of stakeholders in decision-making. For example, the OECD and the G7 are founding members of a growing Global Partnership of AI that emphasizes human-centered AI, human rights, and international collaboration through multi-stakeholder digital inclusion frameworks. The UN Secretary-General has published a roadmap for global digital cooperation (UN, 2020), and the Council of Europe's committee on AI (CAHAI, 2021, p. 2) uses broad multi-stakeholder consultations to examine “the feasibility and potential elements of a legal framework” for AI development and deployment.

There is a similar trend in research papers on AI governance. Tentative AI governance frameworks generally present stakeholder involvement, cooperation, and collaboration as key elements in developing and improving governance procedures, often in combination with multi-level conceptual systems that contain elements such as design principles, value goals and principles, impact and risk assessment procedures, standard- and rulemaking, and oversight (Sigfrids et al., 2022). These frameworks do not elaborate much on practical techniques to involve stakeholders, but they call for greater stakeholder participation mainly by reference to public consultation and deliberation, various design methods, or representative panels and committees (Gasser and Almeida, 2017; Winfield and Jirotka, 2018; Yeung et al., 2019; Reddy et al., 2020; Wirtz et al., 2020; Stix, 2021). Based on the research on developing public AI governance, there however seems to be a need for novel tools and methods to support inclusive and participatory decision-making.

Technological innovations in citizen participation, so-called “civic tech,” may provide AI governance discussions with new practical tools to support the formation of shared understandings in civil society. AI-labeled technology alongside other information and communication technology can foster deliberative and participatory decision-making (Savaget et al., 2019; Arana-Catania et al., 2021). AI tools can potentially improve democratic processes and enhance democratic responsiveness and accountability if they align with social and political changes and values supporting the change (König and Wenzelburger, 2020; Buhmann and Fieseler, 2022). For example, Lee et al. (2019) propose a participatory framework called WeBuildAI for human-centered, algorithmic decision-making. Drawing on collaborative governance and social choice theory, the model proposes a human-centered way to translate individual beliefs into algorithmically represented decision-making patterns, which would be aggregated collectively to represent stakeholders and support decision-making. The framework would thereby “enable people to design an algorithmic policy for their own community” (p. 3).

Poblet et al. (2019) have compiled a list of 130 existing software tools, apps, platforms, and portals designed for civic engagement and participation. They propose that such tools, or ecosystems incorporating them, are most actionable when they are aligned with decision-making institutions. For example, Iceland in 2011, and Mexico in 2016, developed draft constitutions through collaborative editing tools and crowdsourcing, but the processes did not translate into legislation as they were halted when a wider range of administrative institutions got involved. Taiwan has been more successful in aligning civic tools with decision-making institutions and processes (Poblet et al., 2019). Launched in 2014, the vTaiwan project (Hsiao et al., 2018, p. 1) is “an open consultation process that brings the Taiwanese citizens and the Taiwanese government together to craft country-wide digital legislation” with the help of collaborative, open-source engagement tools, such as pol.is, crowdsourcing, and open consultation. It employs a bottom-up process that includes proposal, opinion, reflection, and finally legislation stages. Hsiao et al. (2018, p. 3) reported in 2018 that “26 national issues have been discussed through Taiwan's open consultation process, and more than 80% have led to decisive government action.”

## 4. Toward a systemic and inclusive approach to human-centered AI development

Based on the discussion in this article, we outline the human-centered development and use of AI as a wide socio-technical challenge that requires a systemic governance approach that considers citizens as participating agents. By a systemic approach, we call attention to the interconnectedness of the different actors and technologies in socio-technical assemblages that contribute to producing the systemic output (Meadows, 2008) characterized here as the impacts of AI deployment in society. By accounting for and balancing the different perspectives, interests, and values of actors in society with the various long- and short-term impacts of AI deployment, public administrations can build foundations for socially sustainable governance procedures. The assumption is that a systematic approach based on citizen and stakeholder engagement and inclusion would both strengthen general trust in AI systems and their governing institutions, and improve collective decision-making in AI-related policies, enabling public administrations to support the technology development for the common good.

**Figure 1:**
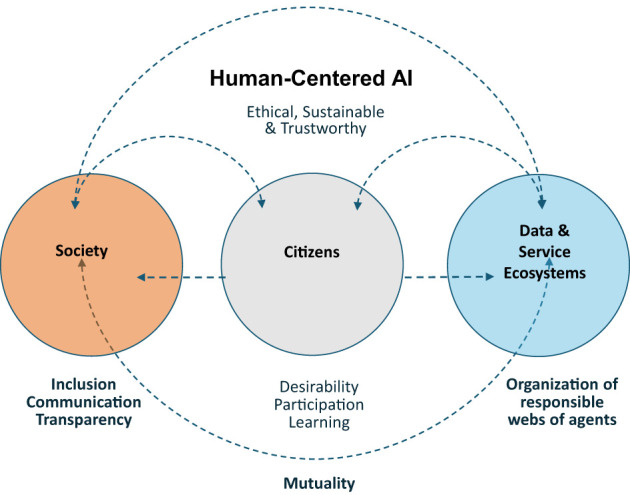
A systemic approach to human-centered AI development and deployment.

Drawing together the arguments in this paper, Figure 1 depicts our perspective on how HCAI could be perceived as a governance perspective that supports emancipatory technology development. The figure shows how the systemic approach to human-centered AI development supports *sustainable and inclusive* societal development, which is founded on *transparency and communication* in decision-making. AI development and deployment should lean on the idea of *mutuality* between public authorities and different agents in data and service ecosystems which can be conceived as responsible webs of agents (Koskimies and Kinder, 2022). Governmental actions related to AI should foster and facilitate societal discourse on the *desirability* of AI, including the active *participation* of citizens, and promote *learning* and understanding of AI and AI ethics.

Collaborative and civic tech tools can help operationalize the systemic and inclusive approach as they enable large-scale engagement and participation in decision-making processes. Stakeholder and citizen engagement and participation technologies have the potential to change the nature, imaginaries, and expectations of both participatory and deliberative decision-making, and the mutual desirability of technology. Literature and case studies in civic engagement provide concrete (software) tools and methods for wide deliberation, mass participation, and methods to aggregate and process preferences in ways previously not possible. Whereas, such engagement tools have, despite their promise, generally failed in the past to affect basic democratic processes (Bastick, 2017), novel AI governance frameworks might well consider the potential and limitations of such technologies in increasing the actionability of ethical principles and facilitating socially sustainable, human-centered governance of AI.

## 5. Discussion

The challenges of governing human-centered AI concern not only how AI should be governed but also the governance modalities themselves (Kitchin and Dodge, 2011; Viljanen, 2017; Weber, 2018). There is a need to move from hierarchical governance and decision-making processes to new forms of inclusive governing that invoke questions regarding the rationale, accountability, and transparency of decision-making, and even pave the way for new stakeholder and citizen engagement (Sigfrids et al., 2022) and participation technologies.

As we have suggested, there is a danger that the emancipatory role of technological development will be forgotten or sidestepped in current governance guidelines and strategies that steer AI-related development and deployment practices. AI policy papers and strategies should consider the wider socio-technical, political, and psychological whole (e.g., Geels and Schot, 2007; Crawford, 2021; Frey et al., 2021) and the ideal of emancipation and enhancing wellbeing, instead of merely respecting moral minimums (Canca, 2019). It is not enough to understand how AI is developed. To create sensible governance models, we also need to reflect upon why and under what conditions AI is being developed in light of its potential impacts on communities and societies. If these aspects remain marginal, AI governance and decision-making will remain superficial from the viewpoint of technological emancipation. This approach also means reflecting on what kind of multidisciplinary expertise is needed (now and in the future) and how trust in AI can be fostered. Deficiencies in building trust and multidisciplinary communication undermine both the adoption of AI technologies and the realization of their potential for the benefit of humanity.

We should note that if the human-centricity concept is understood in terms of its sole and primary focus on humans, it leads to a very limited understanding of both the conditions of life and society, and the human psyche, culture, values, and wellbeing. Human-centricity in the light of the emancipation viewpoint should also be community- and society centered, and include consideration of the natural environment and of other living beings that are part of the planetary and human ecosystems. Planet-centricity could be a more suitable concept for future ethical discussions about aligning AI development and deployment with the UN Sustainable Development Goals. Unfortunately, we are not able to consider this at length here. However, the connections between the systemic approach to human-centricity suggested in this article and conceptualizations of planet-centricity provide an important perspective, one that might be elaborated on in a subsequent article.

Emphasizing the role of citizens as active co-developers in public governance instead of merely users of AI is a paradigmatic shift concerning human-centricity and is necessary for building trust in AI deployment in society. A *systemic approach* to human-centricity is needed to embrace and actualize the emancipatory goals of human-centricity in current AI governance mechanisms. Such an approach could be supported by novel software solutions to enhance design, deliberation, and collaboration processes. The systemic approach requires a re-imagination and novel adaptations of processes and technologies for participation and design in a context where public AI governance is required to be flexible enough to adapt to complex and dynamically changing situations. It requires promoting enlightened communication, mutuality, inclusiveness, and transparency in dynamic processes that integrate society, citizens, and the various data, service, and planetary ecosystems, all of which embrace the principles for ethical, sustainable, and trustworthy AI outlined in this article.

## Data availability statement

The original contributions presented in the study are included in the article/supplementary material, further inquiries can be directed to the corresponding author.

## Author contributions

AS is the main author of the text. All authors listed have made a substantial, direct, and intellectual contribution to the work and approved it for publication. The order of authorship reflects the relative extent of contribution.

## Footnotes

^1^Sometimes also abbreviated as HAI (see for example Stanford University's institute for human-centered AI: https://hai.stanford.edu/).

^2^We use the term user-centeredness in our article to refer to viewpoints rooted in human-centered design traditions. This is a conscious choice, by which we hope to overcome the stumbling block of not being able to clearly articulate differences between HCAI and HCD perspectives.

^3^See especially sections considering European Commission (2020), and the national AI strategies of Korean (2016); Germany (2018); Finland (2019) and Lithuanian (2019).
